# Schizophrenia as a Disorder of Communication

**DOI:** 10.1155/2013/952034

**Published:** 2013-05-12

**Authors:** Margaret A. Niznikiewicz, Marek Kubicki, Christoph Mulert, Ruth Condray

**Affiliations:** ^1^VA Boston Healthcare System, Boston, MA 02301, USA; ^2^Harvard Medical School, Boston, MA 02115, USA; ^3^Brigham and Women Hospital, Boston, MA 02115, USA; ^4^Department of Psychiatry and Psychotherapy, University Medical Center Hamburg-Eppendorf, 20246 Hamburg, Germany; ^5^Department of Psychiatry, LMU Munich, 80331 Munich, Germany; ^6^University of Pittsburgh School of Medicine, Pittsburgh, PA, USA

The first characterizations of schizophrenia invoked the concept of disordered thought and broken mind as central to its clinical presentation [1, 2]. Interestingly, Bleuler's characterization of schizophrenia was couched in terms of four A's association, with its focus on disordered language, affectivity, ambivalence, and autism, all of which implicate different aspects of social function [3]. Bleuler captured much that is still relevant to the study of cognitive dysfunction in schizophrenia and, in fact, covers quite well the topic of social communication dysfunction highlighted in this issue. The research that followed these early characterizations firmly established the link between abnormal brain structure and function, mediated by genetics, and many clinical and cognitive manifestations of this devastating disease [4–6]. Over the last several years, great progress has been achieved in the understanding of mechanisms of schizophrenia [7–10]. And while a comprehensive theory of schizophrenia is still elusive, many compelling accounts of schizophrenia pathology have been put forward and generated valuable insights [8, 9, 11–15]. 

Within the field of study of the cognitive dysfunction in schizophrenia, research has focused on different aspects of what was described recently as “cold cognition” and included attention, memory systems that vary in duration, capacity, and operations, as well as language and perceptual mechanisms [9]. The last few years brought a welcome broadening of this field of study as it has been noted that abnormalities in “hot cognition” including abnormalities in emotion and affect processing from both face and voice are an important component of schizophrenia pathology [16, 17]. Social cognition, whose functions draw on both processes of “cold” and “hot” cognition, has become a focus of intense interest with studies addressing the ability to convey one's attitudes and intentions and adaptively predict and interpret the attitudes and intentions of others. Finally and importantly, it has been increasingly recognized that impairments in social cognition contribute to both clinical and functional outcomes in schizophrenia [18, 19]. 

The aim of the current issue is to broaden the discourse on the nature of cognitive dysfunction in schizophrenia. We propose that the cognitive dysfunction in schizophrenia should be conceptualized as a disorder of communication rather that of language itself and that communication disorder is the core clinical deficit of schizophrenia. We believe that an array of sensory and cognitive processes and their interactions enable human beings to enter into meaningful social communication. Thus, communication in a human society involves a complex set of behaviors that include both formal languages embodied in the rules of phonology, grammar, syntax, and semantics, as well as behaviors that allow conveying emotional states and attitudes, and finally and importantly successful interpretation of these behaviors in others. They depend on effective perceptual processes on one hand and on the successful recruitment of intact higher order processes such as working memory, attention, inhibition, and response selection on the other hand. We would like to argue that neither the focus on the study of language nor the study of social cognition fully captures the communicative difficulties that patients with schizophrenia encounter. Rather, a schizophrenia sufferer is confronted with a poor ability to effectively use language and a poor ability to deploy other communicative devices to achieve successful functioning in a society, both in social and professional settings. Given the complexity of the behaviors under consideration, most studies on cognitive impairment in schizophrenia tend to adopt one of the two perspectives. Thus, the studies are conducted either within the framework of “cold cognition” and focus on the study of language, executive function, and perception or within the framework of “hot cognition” and focus on the study of emotion, theory of mind, and agency, to name a few topics. 

Articles in this issue reflect both perspectives: the focus on abnormal language function as a central characteristic of schizophrenia pathology on one hand and the conceptualization of impairment in schizophrenia as a result of abnormal processes of social cognition on the other. M. A. Boudewyn and colleagues examine language impairment in schizophrenia in a review paper and argue that the extent of language processing difficulties is a function of the complexity of a linguistic message: the more complex the message, the more impairment will be observed. According to this conceptualization, schizophrenia patients should be most impaired in the processing of discourse that calls for the manipulation and reconciling of multiple sources of information. Conversely, they should be least impaired in the processing of single words and word pairs. The authors articulate their proposal of language impairment within the framework of domain general control mechanisms as mediated at the brain level by dorsolateral prefrontal cortex (DLPFC) and anterior cingulate cortex (ACC). They argue that it is these impaired mechanisms that mediate most severe language dysfunction in schizophrenia. This proposal is distinctly different from the hypothesis that language abnormality in schizophrenia is primarily rooted in abnormal processes within semantic memory [15]. As the authors suggest, the hypothesis of prefrontally mediated language dysfunction promises to be a rich source of experimental approaches that will test how different levels of language complexity map onto the degree of dysfunction in the prefrontal systems, how well the results of nonlinguistic tests of cognitive control correlate with the results of language tasks that purport to rely on cognitive control functions, are how tests probing semantic memory processes integrity compared with language tests of context building in terms of observed effect sizes as tested in the same subject group and using the same methodology. It will be important to use approaches that include multiple methodologies as each one—behavioral, event related potential (ERP) and functional magnetic resonance imaging (fMRI)—offers unique and nonredundant pieces of evidence on how language processes are implemented in the living brain and what it means for the theory of schizophrenia. 

The article by S. M. Arcuri and colleagues fits very nicely within the proposal of examining how complex linguistic messages are processed by brain systems. The authors explore language processing in the healthy comparison subjects and patients with and without a formal thought disorder. Drawing on the tradition of ERP studies of language processing in schizophrenia, the authors examine the processing of sentences with congruent and incongruent endings reasoning that the contrast between the sentences that require integration of context relative to those requiring the suppression of inappropriate semantic material will highlight a role of brain regions involved in the inhibition of automatically primed stimuli. The results suggest that the left middle frontal cortex is activated more in the incongruent relative to congruent sentences in the healthy comparison group. When the same incongruent/congruent contrast is analyzed in patients with the formal thought disorder, reduced activation in the left inferior/middle frontal gyri and in the anterior cingulate is reported. Thus, these results seem to support the idea that indeed the brain regions involved in context maintenance and manipulation and in the inhibition of inappropriate semantic entries are involved in the disordered language in schizophrenia. 

Finally, D. Ketteler and colleagues present a new tool to assess the nature of compromised language function in schizophrenia: higher order linguistic function test (HOLF). They also adopt the premise that more complex language forms will create special difficulties for schizophrenia sufferers. However, in contrast to M. A. Boudewyn and S. M. Acruri proposals, the authors focus on ambiguity in language as brought about by single words such as antonyms, synonyms, homonymy, and as well as on interpreting popular adages. While easier operations such as interpreting adages and antonyms were not impaired in the patient group, tasks associated with more complex forms distinguished between the two groups and were correlated with symptom ratings as measured by PANSS scores. These results suggest that complexity of linguistic material does not have to be related to discourse or sentence structure but can be also related to conceptual ambiguity in order to tap into linguistic difficulties in schizophrenia patients. 

The articles by A. P. Pinheiro, H. Fatouros-Bergman, and C. G. Wible are conceived within the framework of conceptualizing schizophrenia as a disorder of social cognition. H. Fatouros-Bergman and colleagues describe a negative facial affectivity bias in patients with schizophrenia that seems to persist across several temporal measuring points. A. P. Pinheiro and colleagues draw attention to the fact that in spite of evidence of affect processing difficulties in schizophrenia, the conscious ratings of emotional valence seem to be intact. The review paper by C. G. Wible advances an argument on the centrality of social communication abnormality in schizophrenia. Rather than focusing on language as a disembodied communication devise, this approach situates language within the context of body-based gesture system whose communicative capabilities are richer than those possible to be achieved within language as a semantic and syntactic system only. As C. G. Wible points out, a live conversation involves not only parsing out words and paragraph meanings, but also relies on correct interpretation of facial expressions, and tone of voice, social salience, agency (who is doing the talking), and intention. An ability to anticipate another person's actions and represent another point of view, often referred to as a theory of mind, is also essential for successful communication. Finally, two major features of schizophrenia pathology, hallucinations and delusions, involve, in addition to abnormalities in cognitive control [20, 21] and in perceptual and attentional processes [22], also abnormalities in the sense of agency [23]. As the article argues, the brain region that supports most of these functions is the temporal parietal occipital junction (TPJ) with projections to inferior frontal regions, hippocampus, and insular regions. Like the proposal put forth by M. A. Boudewyn et al., the hypothesis formulated by C. G. Wible has a promise of generating interesting experimental paradigms to test the role of TPJ in mediating different aspects of social communication abnormality in schizophrenia. Given the differences in the emphasis between the two proposals, the importance of temporoparietal regions articulated by Wible, and the importance of prefrontal regions articulated in the theory of domain general control impairment, these two conceptualizations may be viewed as two competing views of schizophrenia dysfunction. However, they can be also viewed as two complementary views that together describe the phenomenology of schizophrenia better than each of these theories alone. The theory of abnormal control mechanisms provides a compelling account of how a complex message system with its symbolic and multilayered semantics and syntax can be affected by impaired capacity to manipulate its different elements. The theory of abnormal social communication provides a novel perspective on how a formal language may interface with social communicative devices (gestures, emotional facial expressions, tone of voice (prosody)) and faculties (theory of mind, sense of agency, and intention). Thus, together with the already existing theories of abnormal processes within semantic memory largely borne out of priming studies [15] and a theory of perceptual dysfunction in schizophrenia relying on evidence derived from studying sensory auditory and visual processes [11], the theoretical perspectives espoused in the two review papers add to the richness of theoretical conceptualizations of cognitive dysfunction in schizophrenia. 

Overall, this selection of papers is a good representation of the richness of approaches applied to the study of communication dysfunction in schizophrenia. They illustrate the centrality of language dysfunction in schizophrenia as well as the importance of abnormalities in nonlanguage-based semiotic systems as contributors to the schizophrenia sufferers' inability to effectively engage with the world in an act of communication.

*Margaret A. Niznikiewicz* *Margaret A. Niznikiewicz* *Marek Kubicki* *Marek Kubicki* *Christoph Mulert* *Christoph Mulert* *Ruth Condray* *Ruth Condray*
